# Supplementary material for: Are carbon emissions trading and green financial instruments synergistic? -Comprehensive quantitative research based on content analysis
Source: PLoS One. 2024 Mar 7;19(3):e0298601. doi: 10.1371/journal.pone.0298601 (PMC10919601; doi:10.1371/journal.pone.0298601)
Supplement: S1 Data — (ZIP) [file pone.0298601.s001.zip › Data/地级市Z_F工作报告.zip!/║■▒▒/╨ó╕╨╩╨/└┤╫╘╣1⁄2╓┌║┼í╛┬φ┐╦╩2╛▌═°í┐_2016.docx]

各位代表：

　　现在，我代表市人民政府向大会报告工作，请予审议，并请各位政协委员和列席人员提出意见。

　　一、“十二五”工作回顾

　　“十二五”时期，是孝感极不平凡和取得显著成就的五年。面对艰巨繁重的改革发展任务和经济下行压力，在省委、省政府和市委的坚强领导下，在市人大、市政协的监督支持下，市政府紧紧依靠全市人民，攻坚克难抓改革，综合施策促发展，远谋近施增后劲，倾心尽力惠民生，经济社会保持持续健康发展，“十二五”主要目标任务圆满完成（见附件一），大力推进“五个跨越”、加快建设“五个城市”迈出坚实步伐。

　　这五年，经济规模成倍增长，综合实力显著提升。坚持把做大经济总量作为政府工作的首要任务，在调整优化结构中保持经济平稳较快增长。地区生产总值每年净增百亿元以上，总量达到1457.2亿元，是2010年的1.8倍。财政总收入169.4亿元、地方公共财政预算收入122.8亿元，分别是2010年的2.9倍、3.6倍。工业在逆境中增量提质。以“三大工程”为抓手，创新服务方式方法，规模以上工业企业每年增加100家以上，总数达到1257家，增加值719.6亿元，是2010年的2.5倍。高新技术产业增加值167.5亿元，是2010年的2.6倍。拥有中国驰名商标13件、国家地理标志保护产品13个。建筑业产值436亿元，是2010年的3.3倍。农业在调整中取得突破。以“三农三化”为方向，建基地、抓加工、创品牌、增效益，农产品加工产值1360亿元，是2010年的3.6倍，主要农产品稳定增长。完成水利投资116亿元，农村安全饮水普及率达到98.5%。完成土地整治投资41.5亿元，建设高标准农田219.7万亩。农机化率达到73%。防灾减灾体系健全完善，战胜连续四年干旱。减少贫困人口22.3万人。连续五轮“三万”活动获省委、省政府表彰。第三产业在培育中升级发展。以“三个加快”为目标，积极培育现代物流、文化旅游、电子商务等新兴业态，社会消费品零售总额797.1亿元，是2010年的2.1倍。全市金融机构增加到43家，涉农贷款和中小企业贷款余额分别增长2.5倍、3倍。旅游人数1840万人次、综合收入115亿元，分别是2010年的2.3倍、2.5倍。电力、烟草、邮政、通信、石油、盐业等行业实现新发展。投资支撑作用明显增强。滚动实施牵动孝感发展全局的100个重大工程项目，完善“双包保”和“六个一”推进机制，完成全社会固定资产投资1824.9亿元，是2010年的3.2倍，实施亿元以上项目1802个，其中过10亿元项目96个。争取国家级试点22项、省级试点39项。市本级实战主体作用充分发挥。创新市直“三区”体制机制，强化统筹功能，下放113项市级行政审批事项。市高新区升格为国家高新区，高新技术企业达到60家，地方公共财政预算收入突破10亿元，是2010年的6.1倍，产业新城、城市新区的主战场作用凸显。市临空经济区纳入全省大临空规划，基础设施、产业项目、城市新社区三大建设全面推进，临空新城形象初显。市双峰山旅游度假区20里生态画廊形成，华中旅游品牌影响力扩大。县域经济竞相发展。7个县市区生产总值、固定资产投资都超过百亿元，财政总收入都超过10亿元。

　　这五年，统筹城乡步伐加快，城乡面貌显著变化。坚持“全域孝感”理念，推动新型城镇化扩容提质，促进农业转移人口市民化，城镇化率提高到53.2%。市县乡村四级规划全面贯通。市、县总体规划完成修编，“1531”规划空间发展体系形成。孝感中心城市提档升级。孝感城区实施城建项目504个，完成投资241.9亿元。新建改造骨干道路42条，综合提升背街小巷64条，新增绿地面积450多万平方米，新增公交线路8条，建成八一水厂和2座污水处理厂。建成区面积达到55平方公里，人口增加到50万人。县市城区建设力度加大。汉川城北、应城城南、云梦城东、安陆河西、大悟高铁、孝昌澴西等六大城市新区加快建设，全市城镇建成区面积达到252平方公里。“美丽孝感”建设有序推进。“四城同创”、“一线一点”示范成效明显，孝感、应城顺利通过国家园林城市考核验收，孝感荣获省级文明城市五连冠，汉川、应城、安陆进入省级文明城市行列。市、县城区建立市场化、专业化城市综合管理新模式。两个“六张网”不断向农村延伸覆盖,新建通村公路4200公里，延伸供水管网近万公里。“两型”社会建设取得新进展，大别山试验区绿色能源产业发展迅猛。王母湖、野猪湖等湖泊保护取得阶段性成效。“绿满澴川”行动扎实开展，植树造林20万亩，森林覆盖率达到18.9%，建成省级宜居村庄55个。大气污染综合防治力度加大，重点企业污染源在线监控实现全覆盖，饮用水水质稳定达标，主要污染物排放量完成目标任务。

　　这五年，改革开放不断深化，发展活力显著增强。坚持把改革创新、扩大开放贯穿于经济社会发展全过程，发展新动能得到汇聚。通过清障松绑激发市场活力。大力推进简政放权、放管结合、优化服务，市级行政审批事项减少到257项。落实减税降费政策，每年减轻企业负担近3亿元。推行工商注册登记便利化，新增市场主体20.7万户，总数达到31.3万户。最大限度释放技术、人才潜能。积极推进军民融合发展，4个军民结合产业园聚集企业63家，产值达到165亿元。建成省级科研平台40家，设立院士专家工作站50家。建立孵化器13家，孵化企业456家。孝感连续五届荣获国家科技进步城市、获批国家知识产权试点城市。“三情”结合破解发展难题。实施“十大融资办法”、“七大土地增容措施”、“槐荫人才计划”，有效破解融资、用地、人才难题。发挥财政资金杠杆作用，撬动银行扩大信贷84亿元。市、县普遍建立政府性融资平台，发行企业债券94.3亿元，58家企业在创业板、“新三板”和四板挂牌。开工建设“三类”新社区211个，多途径盘活存量土地5.4万亩。招硕引博289人，“三支队伍”建设经验在全省推广。以产业招商提升引资质效。紧盯行业龙头，配套延伸产业链条，

　　突出二次招商，引进产业项目1425个，占项目总数的74.2%，新引进行业龙头企业30家。新批外商直接投资企业120家，新增有出口实绩企业151家，产品出口到137个国家和地区。

　　这五年，社会建设扎实推进，人民生活显著改善。坚持把改善民生作为根本目标，市县乡村四级联动为民兴办实事，民生支出占财政总支出的75.2%。城镇和农村常住居民人均可支配收入分别提高到25700元、12700元。促进充分就业的机制得到完善。积极开展国家创业型城市创建工作，建立城乡统一的就业服务平台，持续开展“就业创业在家乡”活动，支持以创业带动就业，城镇新增就业27.4万人。覆盖城乡的社会保障制度基本建成。“五项社保”参保人数达到466万人次，被征地农民社会保障制度出台实施。城镇居民医保参保率、新农合参合率分别提高到99%、99.9%，大病保险全覆盖，报销比例提高到70%。建成社会福利中心、残疾人康复中心等服务设施141个，发放社会救助资金37亿元，常年救助困难群众41万人。建成保障性安居房4.5万套，改造棚户区13万户、农村危房3.54万户。住房公积金实缴人数增加到21.5万人。均等化的公共服务惠及更多人群。改扩建标准化中小学校301所、幼儿园181所，建成农村教师周转房3200套，资助贫困生37.4万人次。全市整体通过义务教育均衡发展国家认定。湖北工程学院、湖北职院办学水平提升，湖北工程技师学院获批组建。新改扩建市县医疗机构32个、乡镇卫生院77个、标准化村卫生室635个。普遍实行基本药物制度，6个县市全面启动县级公立医院综合改革。卫生计生资源有效整合，出生人口性别比下降6.67个比值。城乡文体基础设施提档加速，“村村通”“村村响”“户户通”等惠民工程如期完成。承办重阳与亚洲孝道文化国际论坛、中国孝廉文化研讨会等活动，每年举办一届中华孝文化旅游节，荣膺“中国孝文化之乡”。全国未成年人思想道德建设先进城市通过复核。安定有序的社会治理格局日益完善。建立综治信访维稳反恐“十大机制”，推进城乡警务机制改革，实行“一村（社区）一警”，建成覆盖城乡的网格化管理和社会治安视频监控系统，应急处置快捷高效，平安孝感建设走在全省前列。阳光信访、法治信访、责任信访深度推进，3个“1+X”服务体系全面建立。安全生产责任层层压实，食品药品和产品质量监管面扩大，市场决定价格机制初步形成。双拥模范城创建成效显著。地方志获评全国先进集体。老龄、残疾人事业有新发展，民族宗教、外事侨务、援疆援恩、对台工作取得新进展，国资监管、统计、调查、人防、保密、档案等工作取得新成绩。

　　这五年，法治政府建设力度加大，政府自身建设显著加强。坚持转作风、抓落实、促跨越，着力打造人民满意政府。依法行政迈出坚实步伐。实行政府常务会会前学法，市、县普遍建立法律顾问制度。坚持依法履职，制定市级行政权力清单、责任清单，建立完善六个工作委员会，对重大事项实行集体议事决策。严格控制机构编制和财政供养人员总量。每年向市人大报告、向市政协通报政府依法行政工作，市政府班子成员领办人大代表建议、政协提案形成常态机制。圆满完成“六五”普法任务。行政效能明显提升。打通政务服务平台，下沉服务事项300项。深化“三集中三到位”改革，审批程序简化、时限压缩。扎实开展党的群众路线教育实践活动、“三严三实”专题教育，“四风”整治持续深化。践行“一线工作法”，强化主责担当，一支政治强、责任清、作风正、工作实的公务员队伍正在形成。廉洁从政自觉性增强。推行同类项目整体打包招标建设，实行规划审批前公开公示制，建立重大项目质量终身负责制，对政府性投资项目全程审计，对财政性资金和重大项目全过程监管，让权力在阳光下运行。严格落实党风廉政建设主体责任，坚持把纪律挺在前面，建立完善警示教育、谈话提醒、问责追责制度，持续推进纠风正纪促跨越活动。支持监察部门履行监督责任，对不作为、慢作为、乱作为进行查处问责。

　　刚刚过去的2015年，实体经济遇到前所未有的困难，我们坚定信心，主动作为，全力对冲下行压力，经济保持平稳健康的发展势头，较好完成了市五届人大六次会议确定的各项任务（见附件二）。

　　一年来，我们致力于稳增长壮产业，实体经济稳中向好。把帮扶实体经济作为稳增长的主要抓手，狠抓中省重大政策部署落地生根。扎实开展“优质服务办成事、创新创业干成事”和“千名干部进千企”活动，组织开展“五个对接”，创新财政资金使用办法，精准服务企业，设立10亿元市级产业发展基金，在25个产业集聚区建立院士工作站，政府与企业携手共渡难关。引导电子商务与实体经济深度融合，实现线上销售额83亿元。大力推进农业产业化，推动农业与文化、旅游、休闲体验相结合，5.6万人实现精准脱贫。

　　一年来，我们致力于扩投资增后劲，发展基础不断夯实。把项目投资作为推动发展的生命线，精准对接中省战略规划，推动重大战略项目化、具体化，策划“十三五”项目库、总投资3.8万亿元，谋划106个项目向社会推介，争取中省预算内投资项目491个、资金24亿元。推动重大项目加快建设，100个重大工程项目完成年度投资410亿元，汉十铁路、武汉城市圈环线高速、鄂北水资源配置工程孝感段等一批重大项目开工建设。推动招商项目向产业集聚、向园区集中，20余家日资企业扎堆发展。

　　一年来，我们致力于抓改革促创新，市场活力加速释放。把改革创新作为推动发展的第一动力，以激发市场活力、减轻企业负担、破解融资难题为着力点，深入推进行政审批等各项改革。强化抓创新就是抓发展、谋创新就是谋未来的理念，出台创新创业“1+2+N”支持政策，设立5000万元投资引导基金，发放1000万元科技创新券。组建科技众创联盟和各类众创空间，搭建“中华孝道创业园”“创业大学”等创新创业平台，涌现出一批充满活力的创业团队和市场主体。

　　一年来，我们致力于抓统筹重生态，城乡面貌进一步改善。以绿色发展为引领，围绕建设生态、宜居、美丽孝感，推动实施一批道路、供排水、无害化处理等基础设施，完善城镇功能，改善城乡生产生活条件。推动实施一批绿化亮化、环境洁净工程，孝感城区改造提升“六大生态主题公园”，建成3条城市防护林景观带，在市域内建成王杨线、应汤线、观音湖线等7条绿色长廊。推动实施一批生态环境保护工程，朱湖、府河、汈汊湖、老观湖等国家级湿地公园生态得到修复。全省新农村建设暨城乡一体化工作会议在我市召开，孝感经验在全省推广。

　　一年来，我们致力于惠民生兜底线，人民群众幸福指数得到提升。以普惠共享为根本目标，增加公共服务、公共产品供给，努力让发展成果惠及更多人群。真心实意帮扶特殊群体，通过“双助”行动实现2271名困难人员就业创业，五保供养标准、低保保障线进一步提高。为孝感城区60岁以上老年人全部购买意外伤害综合保险，为14.7万名留守老人建立健康档案，对7.66万名留守儿童实施“六有”关爱。创新方式，结合去库存，筹集800套房源，解决孝感城区困难家庭住房问题。着力推动教育、卫生、文体等社会事业均等化，全市新增校车582台，中小学校严格执行划片招生、就近入学，市直7对学校建立校际联盟。卫生计生信息服务实现“一卡通”，实施分级诊疗，推进医养结合。集图书馆、博物馆、群众艺术馆等六大场馆于一体的市文化中心主体工程封顶。统筹推进综治、信访、维稳和安全生产，形成和谐稳定的大平安格局。

　　各位代表，五年来取得的成绩来之不易。这是省委、省政府和市委正确领导的结果，是全市人民共同奋斗的结果，是社会各界大力支持的结果。在此，我代表市人民政府，向辛勤工作在各行各业的广大干部群众，向各民主党派、工商联、人民团体、各界人士和离退休老同志，向驻孝部队、武警官兵和中省单位，向所有关心支持孝感发展的同志们、朋友们，向在澴川大地不懈奋斗的创新创业者，表示衷心的感谢和崇高的敬意！

　　五年来积累的经验难能可贵。在政府工作实践中，我们体会到：必须坚持把大力推进“五个跨越”、加快建设“五个城市”作为科学有效路径，解放思想，敢于担当，抢抓机遇，加快发展；必须坚持打基础、利长远，扭住工业这个“牛鼻子”，抓住项目这个“压舱石”，突出新型城镇化这个主抓手，远谋近施做大经济总量；必须坚持改革创新谋发展，扩大对外开放，激励创新创业，用改革、创新和市场的办法破解难题；必须坚持维护社会公平正义，带着感情办好民生实事，努力让人民群众共享发展成果；必须坚持“一线工作法”，“一对一、实打实”服务市场主体，营造更加宽松的发展环境；必须坚持用制度管人、管权、管事，切实做到秉公用权、依法行政，从源头上打造廉洁政府。

　　在总结成绩的同时，也要看到政府工作的不足和差距。主要是：经济发展的质量和效益不够高，产业结构不优，新技术、新产业、新业态培植的力度还不大，产业、区域、城乡融合发展的水平有待提升；绿色发展步伐不快，转型发展的任务重，面临着保持“中高速”增长与保护环境的双重压力；公共服务供给能力不强，市场化、社会化的手段运用不多，与满足群众多样化、个性化的需求还不相适应；少数公务员服务市场主体、服务人民群众的能力和作风还有较大差距，法治政府建设任务紧迫而艰巨。对于这些问题，我们将不回避、不懈怠，强化工作举措，积极推进解决。

　　二、“十三五”目标任务

　　各位代表，“十三五”时期，孝感仍处于大有作为的黄金机遇期，是积蓄能量释放期、综合优势转化期、“四化同步”发展加速推进期，我们面临着长江经济带、长江中游城市群、汉江生态经济带、大别山革命老区振兴发展等多重战略机遇；全面深化改革和宏观调控政策的共振效应叠加，发展内生动力增强；经过多年的努力和积累，跨越发展的基础和条件越来越夯实。我们有信心、有决心同全市人民一道，抢抓机遇，担责担难，扎实苦干，奋力实现新作为。根据《中共孝感市委关于制定全市国民经济和社会发展第十三个五年规划的建议》，市政府制定了《孝感市国民经济和社会发展第十三个五年规划纲要（草案）》，提请大会审议。

　　“十三五”经济社会发展的总体思路是：高举中国特色社会主义伟大旗帜，全面贯彻落实党的十八大和十八届三中、四中、五中全会精神，以马克思列宁主义、毛泽东思想、邓小平理论、“三个代表”重要思想、科学发展观为指导，深入贯彻习近平总书记系列重要讲话精神，围绕“四个全面”战略布局孝感实施，全面落实创新、协调、绿色、开放、共享发展理念，坚持“三维纲要”，坚持竞进提质、升级增效，以科学发展为主线，以深化改革、扩大开放、创新驱动为动力，以法治孝感建设为保障，以提高人民群众福祉为根本，以大力推进“五个跨越”、加快建设“五个城市”为路径，加快推进武汉城市圈副中心城市建设步伐，努力打造“湖北的苏州”和“魅力孝感”，确保如期全面建成小康社会，不断开创第三次创业新局面。

　　“十三五”的奋斗目标是，主要经济指标居全省前列，增幅高于全国、全省、武汉城市圈、大别山区平均水平（见附件三）。

　　“十三五”时期，我们将按照五大发展理念，着力发展新技术、新产业、新业态，不断提高发展的质量和效益，推动创新发展；着力推进产业融合、区域融合、城乡融合，积极拓展发展新空间，推动协调发展；着力发展绿色经济、低碳经济和循环经济，建设资源节约型和环境友好型社会，推动绿色发展；着力打造开发园区核心载体，发展创新共同体和平台经济，推动开放发展；着力提高社会事业均等化水平，优化供给方式，提升人民群众生活质量，推动共享发展。通过大力推进“五个跨越”、加快建设“五个城市”，实现建设武汉城市圈副中心城市战略目标。

　　（一）加速推进产业跨越，奋力实现经济强市建设的新突破。坚持速度质量效益并重，提升产业层次，壮大产业实力。以实施中国制造2025孝感行动为抓手，推动工业提速扩容、提质增效。改造提升传统支柱产业，通过兼并重组、智能化改造和新技术、新模式、新业态集成应用，推动食品饮料、纺织服装、盐磷化工三个产业主营业务收入过千亿元。加快发展战略性新兴产业，通过政策集成、要素集聚，推动高端装备制造、光电子信息、新材料、新能源、生物医药等产业集聚发展，打造千亿级军民融合产业集群。推进“两化”深度融合，推动物联网、大数据、云计算、智能机器人等信息技术与制造业结合，加快培育“数字企业”、智能工厂。以“农业强、农民富、农村美”为方向，推动农业种养加一体、“接二连三”融合发展。加快转变农业发展方式，落实藏粮于地、藏粮于技战略，稳步提高农业综合生产能力。有序推进农业规模化经营，深入实施“四个一批”工程，产值过10亿元农业产业化企业达到30家以上。大力发展“互联网+农业”，培植壮大新型农业主体，推进农村三次产业深度融合。以打造区域性现代服务业中心为目标，推动新兴服务业总量壮大、占比提升。加快生产性服务业向专业化和价值链高端延伸，建设市高新区金融港，打造区域性金融中心。推进生活性服务业向精细化和高品质转变，着力发展以“孝感天下、情润山水”为主题的特色旅游，实施社区服务体系工程，满足群众个性化的消费需求。建设智慧城市，加快推进信息惠民工程，培育壮大新兴业态。

　　（二）加速推进城镇跨越，奋力实现鄂豫省际区域性中心城市建设的新突破。落实中央城市工作会议精神，围绕“1531”城镇体系，南依大武汉，北联大别山，城乡大统筹，大力推进以人为核心的新型城镇化。城镇化率达到61%以上。按照“六大组团”布局，加快构筑百万人口大城市格局。坚持新建与升级并重，优化提升主城区核心承载功能，创建国家卫生城市、生态园林城市、环保模范城市，争创全国文明城市。加快建设东城新区行政、文化、商住等功能区，推进生态文化旅游区、南城新区项目建设，建成市临空经济区10平方公里核心区，打造兼具孝文化、楚文化特色的云梦水乡园林城市组团。坚持城乡统筹，加快构建城乡互动、功能互补的城镇体系。科学确定5个县市城区发展定位，拉开中等城市框架，打造县域人口、产业集聚的主战场。推进30个重点镇围绕“一镇一业”，走规模化、差异化发展道路。选择6个重点镇开展小城市试点建设，逐步形成片区发展中心。以100个中心村为示范，建成500个风貌特色显著、产村高度融合、人居环境优美的美丽村庄。围绕推动绿色发展，加快建设生态宜居的美丽孝感。坚持绿色产业与生态保护两手抓，加快发展“两型”产业和大别山试验区绿色产业，推进产业、园区、企业循环化改造，推进节能、节水、节地、节材，推进矿产资源综合开发利用。加强湿地与河湖生态系统的恢复和保护，持续改善空气质量，加强水污染、重金属及危险废弃物治理，全面推进土壤修复，城镇和农村垃圾无害化处理率分别达到100%和95%以上，形成“蓝天、碧水、宁静、绿地、田园”的自然之美。

　　（三）加速推进文化跨越，奋力实现中华孝文化名城建设的新突破。培育和践行社会主义核心价值观，弘扬“至孝至诚、图强图新”的孝感精神，打造孝文化特色品牌，壮大特色文化产业。加强孝文化研究和载体建设，提升中华孝文化名城影响力。推进孝文化与红色文化、楚文化、李白文化等地方特色文化深度融合，打造孝廉、孝德、孝亲、孝诚精神载体；把孝文化厚植在城市建筑景观中，打造一批孝文化主题公园、民俗文化基地等标志性物质载体；整合中华孝文化旅游节、黄香文化节、孟宗文化节等节庆品牌，打造孝文化活动载体，真正让城市成为文化的容器。健全完善公共文化服务体系，满足人民群众日益丰富的精神文化需求。高标准建设市、县博物馆、图书馆、群艺馆，建成上下联动、覆盖城乡的公共文化设施网络。建设各类文化广场，方便群众就近健身休闲。加强文化遗产保护，设立槐荫文艺奖，创作更多文艺精品。加大融合、培育、壮大力度，把文化产业打造成为新的支柱产业。推进传统媒体与新兴媒体融合，增强地方主流媒体传播力、公信力、影响力，唱响主旋律，传播正能量。推动文化与科技、教育、体育、旅游融合，规划建设文化传媒产业园。加快发展养老服务业，让老年人高质量安享晚年，打造具有孝文化特色的休闲养老服务品牌。

　　（四）加速推进动力跨越，奋力实现创新城市建设的新突破。着眼于激发活力，推进重点领域和关键环节改革，拓展发展空间，鼓励支持大众创业、万众创新。进一步明确供给侧改革的主攻方向，最大程度收获改革“红利”。坚持简政放权、放管结合，推动政府职能向营造环境、优质服务、维护公平转变。以激发活力为目标，推进市场化改革，从制度层面为市场主体清障松绑。深化各项改革，抓好改革试点，创造更多孝感经验。牢固树立开放发展的理念，最大维度扩大对外开放。依托武汉自主创新示范区和大临空经济区，以市高新区、市临空经济区和7个省级开发区为载体，深度融入武汉城市圈、长江经济带、长江中游城市群，形成区域发展共同体。以大别山革命老区振兴发展为契机，推动市县交通一体，促进产业错位互补，实现市域协同发展。坚持“六度”原则，招大、引强、选优，创新招商方式，提高招商实效。依托自由贸易区、“一带一路”，鼓励企业开拓新兴市场，设立境外贸易和投资机构。抢抓国家全力支持“双创”的政策机遇，最大限度激发创新创业动能。培育企业家精神，厚植创新创业文化，强化企业创新主体地位。大力实施“槐荫人才计划”，开展“大学生创新创业引领计划”，推进回归创业工程，壮大创新创业群体。鼓励发展众创、众包、众扶、众筹，推动“一校一街一汇十园区”建设，健全创新创业平台。完善激励机制，推进创业带动就业，建成国家级创业型城市和国家知识产权示范城市。

　　（五）加速推进民生跨越，奋力实现人民幸福城市建设的新突破。坚持把更多财力向民生倾斜，着力推进“五个全覆盖”，彰显社会公平正义，有效提升城乡居民生活质量和幸福指数。推进更加高效科学的社会治理全覆盖。加强法治政府建设，完善依法行政制度体系，推进行政决策科学化、民主化、法治化。丰富完善3个“1+X”服务体系，全面深化网格化管理和精细化服务，统筹推进“三类”新社区建设。实施更加严格的食品药品、安全生产监管，坚持综治信访维稳反恐一起抓，创新完善立体化治安防控体系，保持社会和谐稳定。推进更高标准的社会保障全覆盖。实现社保由制度全覆盖向法定人员全覆盖转变，“五项社保”参保突破500万人次。对困难群体实现应保尽保、应助尽助，提高城乡低保、医疗救助、五保供养、临时救助标准，提高养老服务、儿童福利、残疾人保障水平，基本建成适度普惠型社会福利体系。以“精准扶贫、不落一人”为总要求，到2018年底，实现33.95万贫困人口全部脱贫销号、503个贫困村全部脱贫出列、2个贫困县全部脱贫“摘帽”。推进更加公平、更有质量的教育全覆盖。建立统一的城乡义务教育经费保障机制，大力发展普惠性、公益性学前教育，推动义务教育优质均衡发展。普及高中阶段教育，实现更高水平的职业教育，支持高校加快发展。推进更加便捷、实惠的基本医疗卫生全覆盖。建设健康孝感，全面深化医药卫生体制改革，实现公立医院综合改革全覆盖。推进城乡公共卫生计生服务均等化，提高人民群众医疗卫生保健水平。大力普及卫生保健知识，引导健康绿色生活方式。积极应对人口老龄化，促进人口均衡发展。推进更加普惠、公平的住房保障全覆盖。以满足新市民需求为出发点，以有效保障困难群体住房为重点，构建人有所居的多层次住房保障体系。新增保障性住房1.5万套，实施棚户区改造9万户，住房公积金缴存人数增加到28万人。

　　三、2016年工作安排

　　2016年是“十三五”开局之年。我们将围绕工作目标（见附件四），按照“率先、进位、升级、奠基”的要求，坚持稳增长、调结构、惠民生、防风险，着力加强结构性改革，在适度扩大总需求的同时，去产能、去库存、去杠杆、降成本、补短板，重点抓好五个方面工作。

　　(一)强化“三大支撑”，在做大做强实体经济上实现新开局。

　　强化产业支撑。推动工业转型升级。继续实施“三大工程”，启动中国制造2025孝感行动，支持企业通过自主创新提升核心竞争力，优化存量、引导增量、主动减量。全年新增销售过10亿元企业3家、规上企业100家、小微企业1000家。引导关联企业集中连片发展，推动25个产业集聚区扩规提质，新增主营业务收入过百亿集聚区2个，总数达到10个。设立军民融合产业基金，主动对接航天科工等国家军工集团，做强汽车零部件、智能制造产业，推动整车生产引进取得突破。建立惠企政策月度发布制度，及时把中省政策送到企业，服务到企业家。组建产业促进专班，健全“一对一”包保企业长效机制，把中央提出的“六降”政策落实到位，切实把企业的成本降下来。转变农业发展方式。大力推广“一高三新”模式，实行良种良法配套、农机农艺结合、循环有机农业连接。完善农产品加工园区公共服务功能，支持产业化龙头企业发展，推动农产品就地加工转化，农产品加工业产值增长10%。抓好农产品质量安全示范县创建工作，建成市农产品质量安全检测中心，大力发展绿色农业、有机农业。实施农田水利“七大工程”和高标准农田整治工程。做好第三次全国农业普查和气象、水文等工作。补齐三产业短板。增加优质产品、优质服务的有效供给，积极培育扩大信息消费、绿色消费、养老健康消费等新的消费热点。新增限上单位50家。大力发展电子商务，创建省级电子商务示范企业、示范基地，完善农村现代流通网络体系，推进电商下乡、农产品进城，把更多的本地产品销出去。推进金融服务网格化，支持企业上市和债券融资，防范金融风险。综合施策加大去库存力度，保持房地产平稳健康发展。提升重点景区服务功能，建设乡村生态文化旅游示范带，增强“一日游、两日游”短线休闲度假吸引力，旅游综合收入突破128亿元。

　　强化项目支撑。主动对接中省政策和战略，积极争取中省投资在孝感有更大份额。抓紧做好“十三五”项目前期工作，列出项目清单加快推进。集中力量推进牵动孝感发展全局的100个重大工程项目，实施过亿元项目1000个以上。务实推进中、省试点项目，高质量完成年度目标。在公共服务、生态保护、基础设施等领域，推广政府与社会资本合作(PPP)模式，启动建设一批示范项目。加快汉十铁路孝感段、316、107、346国道改扩建、孝感至应城、汉川至蔡甸一级公路等工程建设，力争武汉城市圈环线高速孝感段、麻竹高速大悟段主体工程完工，汉孝城际铁路建成通车。打造“智慧孝感”，启动智慧政务、医疗、教育、交通等工程建设，提升信息惠民水平。

　　强化园区支撑。以园区为平台，用发展新空间培育发展新动力。发挥市高新区“一区四园”的引领作用，补短板、强功能、促发展，引进投资过5000万元项目20个、开工建设20个、竣工投产20个，三大主导产业规模达到120亿元。着力提升市临空经济区核心区承载功能，加快建设已签约落户项目，启动高新技术和文化旅游产业园建设。做实做强市双峰山旅游度假区，提档升级核心景区，加快建设国家级旅游度假区。支持各县市区围绕“十三五”发展定位，实现特色、差异、联动发展。孝南、汉川、应城按照国家级重点开发区域定位，推进科技资源、开放平台集中,重点打造新型产业聚集区、汉江生态经济带开放开发示范区和全国循环经济发展示范区。云梦、安陆按照农产品主产区定位，大力发展农产品加工为主的特色产业，打造城乡一体、“四化同步”示范区。大悟、孝昌按照重点生态功能区定位，依托山水资源发展绿色经济，打造华中绿色能源之都、全国苗木花卉交易基地。

　　（二）实施“三大攻坚”，在突破重点难点上实现新开局。

　　实施结构性改革攻坚。完成好中央、省级各项年度重大改革任务，有序推进政府层面改革。更大力度推进简政放权，开展新一轮市级行政权力和政务服务事项清理，公布市、县两级权力清单和责任清单，全面深化商事制度改革，建立投资项目联合审批平台，提高审批效率。有序推进户籍、财税、金融、企业、价格等重点领域和关键环节改革。深化市直“三区”和7个省级开发区体制机制改革。完成农村土地承包经营权确权登记颁证工作，推进农村产权交易、集体林权、粮食流通和供销合作社综合改革。

　　实施新型城镇化攻坚。突出城市规划的历史传承和区域文化，突出建筑风格的主色调，强化规划的引领性、强制性、严肃性，坚持“多规合一”，一张蓝图绘到底。继续实施国家新型城镇化试点三年行动计划，积极推进“4+2”试点任务。着力配套完善孝感中心城区整体功能，实施地下综合管廊和海绵城市工程，稳步推进旧城和棚户区改造。加强绿化管护，启动航空公园建设，完成垃圾填埋场扩建工程，设置公共自行车便民站点，改造提升背街小巷、物业管理薄弱小区基础设施，完成老城区水改6000户，新增天然气用户2万户。围绕促进城乡协调发展，全面提升县市城区、中心镇辐射带动功能。持续推进“四城同创”、文明卫生村镇和“一线一点”建设，深化城市综合管理，切实加强城市安全、交通、环境等方面的精细化管理，通过省级卫生城市复查，提名国家卫生城市，支持县市争创省级文明城市。继续实施“绿满澴川”行动，加强传统村落保护，启动新一轮美丽乡村建设和农村垃圾综合治理行动，让群众生活得更方便、更舒心、更美好。

　　实施精准扶贫攻坚。把精准脱贫作为重大政治任务，在巩固精准识别成果、解决好“扶持谁”的基础上，有效落实“谁来扶”、“怎么扶”、“如何退”的各项硬措施。实施“五个一批”工程，配套完善“1+N”扶贫措施，整合各方力量、各类资金、各种资源，因地制宜，因户施策，以增强群众自我造血功能为重点，强化自主脱贫内生动力，确保年度9万贫困人口脱贫、153个贫困村出列。

　　（三）打造“三大优势”，在推动要素集聚上实现新开局。

　　打造创新创业优势。以创建国家级创业型城市和国家知识产权示范城市为契机，推动创新引领发展。加大“双创”政策落实力度，整合各类孵化资源，设立种子资金，孵化企业100家。鼓励科技人员、大学生、返乡农民工、非公人士创业，搭建场地、资金、技术、市场等创新创业平台，支持各类主体创新创业。支持湖北工程学院、湖北职院建设科技孵化园、大学生创业园。完善科技成果转化激励机制，让更多成果走出实验室，实现科技成果项目化、产业化。新增高新技术企业20家以上，高新技术产业增加值增长11%。

　　打造对外开放优势。加快推进汉孝融合、市域融合、园区融合，共享试点示范政策，共享招商信息资源，打造区域发展共同体。聚焦优势产业和新兴业态，瞄准行业龙头，精细精准跟踪对接，力争新引进投资过亿元项目70个，其中过10亿元10个。引入第三方评审选商机制，严格把好项目准入关，提高招商引资质效。充分发挥异地商会作用，继续实施回归工程。加强与海关和商检机构的工作对接，提升对外贸易便利化水平。规范外派劳务管理，支持更多企业扩大境外投资。

　　打造生态环境优势。落实主体功能区规划，划定永久基本农田和生态保护红线。推动“两型”社会和大别山试验区绿色、低碳、循环产业发展，推广绿色建筑和建材。强化问责考核，完善政府、企业、公众共治的环境治理体系，建设4个环境空气质量自动监测站。大力实施蓝天、碧水、净土三大工程，集中精力打好大气污染整治、水污染防治、土壤污染治理三大战役，确保省级环保模范城创建达标，完成主要污染物排放量省定目标任务。

　　（四）完善“三大体系”，在推进共建共享上实现新开局。

　　完善公共服务体系。创新就业服务方式，拓宽技能培训平台，加强就业援助，扶持困难群体就业8000人，城镇新增就业4.5万人。大力促进教育公平、优质发展，实施第二期学前教育三年行动计划，提高农村义务教育水平，推动优质学校资源共享，完成“全面改薄”年度项目建设，建成玉泉小学北京路校区，启动建设东城新区401学校。加强乡村教师队伍建设，切实改善乡村教师教学生活环境。实现家庭困难学生资助全覆盖，资助学生6.5万人以上。切实改善群众就医体验，推动市、县优质医疗资源下沉，免费培养100名乡村医生，完成4个县级医疗卫生机构、10个乡镇卫生院、164个村卫生室改造项目，所有村卫生室实现“五化”。推进健康管理、医养结合试点，为留守儿童、空巢老人提供健康服务。落实一对夫妇可生育两个孩子政策，加强妇幼保健能力建设，综合治理出生人口性别比。切实推动文体服务标准化、均等化，全面推进“十星”系列文明创建活动，关心下一代健康成长。办好中华孝文化旅游节和市第五届运动会。市文化中心部分场馆建成投入使用。实施全民健身计划，开展全民阅读活动，引导群众文明健康生活。安装健身器材330套（处），举办演出活动2000场以上。继续做好民族、宗教、外事、侨务、对台等工作。进一步加强国防动员、民兵预备役、人防、编制、统计、档案、地方志、保密等工作。

　　完善社会保障体系。全面实施全民参保登记，完成机关事业单位养老保险制度改革，新增“五项社保”参保10万人次。健全完善临时救助、医疗救助、重特大疾病保险制度，大幅提高城乡低保、五保供养标准。对弱势群体实施分类救助，建立困难残疾人生活补贴和重度残疾人护理补贴制度。提升市社会福利康复中心服务功能，加强农村福利院建设和安全管理，城市社区和农村居家养老服务中心覆盖面分别达到90%和20%以上，孝南、孝昌社区居家养老服务中心实现全覆盖。筹集公共租赁住房2210套，完成棚户区改造3.5万户。推动小区物业管理提档升级。新增住房公积金实缴人数1万人、公积金贷款12亿元。

　　完善公共安全体系。加强食品药品全过程监管，建立企业“红黑名单”制度，建成市检验检测中心。严格落实安全生产责任制，实施安全生产网格化管理，推进道路交通、消防管理社会化，加大对电梯、锅炉等特种设备的专项整治力度，建立安全生产检查清单，实行“双随机”制度，坚决遏制各类安全生产事故的发生。强化网格化管理作用，开展社区“减负增效”专项治理，支持和推动志愿者服务。建立“互联网+法律援助”综合服务平台，成立行业性专业性人民调解委员会，重视社会矛盾纠纷前端治理，推动人民调解、司法调解、行政调解有效衔接、整体联动。深入推行阳光信访，依法依规解决群众合理诉求。落实综治领导责任制，织密防控体系，高压反恐防暴，重拳打黑除恶，压警情控发案，建设平安孝感。建立部门官方微信、网络发言人制度，正确引导网络舆情。健全应急管理体系，有效处置突发事件。

　　民生决定目的。多年来，我们持续为民兴办十件实事，补齐了民生需求中的很多短板。从今年开始，我们将通过市长信箱、媒体征集等多种渠道，常年收集实事项目，直接纳入日常工作，不限数量，应办尽办，让人民群众在共建共享中有更多获得感。

　　（五）提升“三大能力”，在政府自身建设上实现新开局。

　　提升依法办事的能力。出台实施《孝感市法治政府建设规划（2016-2020）》，用法治引领政府工作。围绕城乡建设与管理、环境保护、历史文化保护等领域，开展政府立法工作。启动“七五”普法，大力营造尊法、学法、守法、用法的社会氛围。自觉接受人大法律监督、工作监督和政协民主监督，广泛听取社会各界的意见和建议，高质量办理人大代表建议、政协提案。严格按照法定程序进行决策，完善政府法律顾问制度，落实重大行政决策出台前向本级人大报告、与政协协商制度，实行终身责任追究和责任倒查机制。发挥行政复议监督纠错作用，规范部门执法行为。落实行政机关负责人出庭应诉规定。在市直“三区”和各县市区开发区实行综合行政执法，在市、县城管领域推行相对集中行政处罚权。

　　提升高效做事的能力。建立政务云平台，建成启用市民之家，打造部门联动、联网运行、覆盖到村的政务服务“线上政府”。集约整合基层各类便民服务资源，简化优化服务流程。大力倡导“一线工作法”，每个政府部门确定3-5个重点难点问题，集中精力推动大事难事，形成竞相有为、主动作为的干事创业氛围。加强经常性政务督查，强化监察联动监督，对政府出台的文件、研究的重大事项，实行责任清单制。巩固党的群众路线教育实践活动和“三严三实”专题教育成果，深入整治不作为、慢作为、乱作为等问题，严格治庸问责，确保令行禁止、政令畅通、执行有力。

　　提升干净干事的能力。坚持把纪律挺在前面，强化政治纪律和规矩意识，通过谈话提醒、重点约谈、问责倒逼，把硬约束、强监督、严执纪的措施落到实处。最大程度让政府权力在阳光下运行，公共资源交易项目进平台实现全覆盖，政府投资项目实现全程审计监督，经营性国有资产实现集中统一监管，切实把权力关进制度的“笼子”。严格落实党风廉政建设主体责任，支持监察部门履行监督责任，加强源头治腐，持续深化“四风”整治，强化监督执纪，从严追责查处，以反腐倡廉的实际效果取信于民。

　　各位代表！未来五年是蓝图变成现实的五年。发展全凭实干，奋斗成就梦想。让我们在市委的坚强领导下，珍惜机遇，牢记重托，只争朝夕，担当有为，大力推进“五个跨越”、加快建设“五个城市”，不断开创第三次创业新局面，为全面建成小康社会的宏伟目标而努力奋斗！
